# University of Louisville

**Published:** 1852-04

**Authors:** 


### THE WESTERN JOURNAL.

THIRD SERIES.—VOL. IX.—NO. IV.

LOUISVILLE, APRIL 1, 1852.

### UNIVERSITY OF LOUISVILLE.

At the annual Commencement of the Medical Department of the University of Louisville, held on the evening of the 5th of March, the degree of M. D. was conferred upon ninety-two students of the institution. At the same time, *Crispin D. Boaz*, of Kentucky, a graduate of Hampden Sidney Medical College, and *J. Dickson Smith*, of Georgia, a graduate of Jefferson Medical College, were admitted to the *ad eundem* degree; and the honorary degree was conferred upon *Dr. W. T. S. Cornett*, of Versailles, Ind., *Dr. Alfred W. Hines*, of Bardstown, Ky., and *Dr. Albert P. Wheeler*, of Wheeling, Va. Of the graduates in ordinary, 37 were from Kentucky, 14 from Tennessee, 14 from Mississippi, 8 from Indiana, 7 from Alabama, and the rest from Missouri, Louisiana, Iowa, Arkansas, and South Carolina. Some impressive remarks were addressed to the graduates by the Hon. James Guthrie, President of the Board of Trustees, after which Prof. Miller delivered the Valedictory address in behalf of the faculty. Both these addresses were appropriate and happy, and contained sentiments and admonitions which will often be revived in the recollections of the young gentlemen just embarking in the arduous and anxious duties of professional life. Prof. Miller offered the graduates some seasonable and judicious cautions against quackery, by which, he warned them, they would be courted and if possible cajoled into giving it their countenance and patronage in some of its countless protean forms.

The University of Louisville has again contributed her full annual quota to the ranks of the profession, only two schools in the country, we believe, having contributed more; and our conviction is that her graduates will be found well instructed in the elements of medicine, and as fully prepared for its practice as any similar body who have gone to swell its ranks. But where are all these young doctors to go, and what is to become of them? are questions which some will ask with concern. They have been very well answered in a discourse before the Franklin Medical Society of the State of Massachusetts, by Dr. Stephen W. Williams, from which, for the satisfaction of those who feel a solicitude on the subject, we make a short quotation. "There are in the United States," says Dr. Williams, "about twenty-five millions of inhabitants at the present time. It is supposed that about one physician is required for about one thousand inhabitants, which is a small estimate. This will give twenty-five thousand to the population of the country. If the increase should be as great for the time to come as it has been for a few years past, one million of inhabitants will be annually added to our population, which will require one thousand physicians more a year, at the rate we have estimated, just about as many as now graduate."

